# Current and Calcium Responses to Local Activation of Axonal NMDA Receptors in Developing Cerebellar Molecular Layer Interneurons

**DOI:** 10.1371/journal.pone.0039983

**Published:** 2012-06-27

**Authors:** Bénédicte Rossi, David Ogden, Isabel Llano, Yusuf P. Tan, Alain Marty, Thibault Collin

**Affiliations:** Laboratoire de Physiologie Cérébrale, CNRS-UMR 8118, Université Paris Descartes, Université Paris Diderot, Paris, France; The Research Center of Neurobiology-Neurophysiology of Marseille, France

## Abstract

In developing cerebellar molecular layer interneurons (MLIs), NMDA increases spontaneous GABA release. This effect had been attributed to either direct activation of presynaptic NMDA receptors (preNMDARs) or an indirect pathway involving activation of somato-dendritic NMDARs followed by passive spread of somatic depolarization along the axon and activation of axonal voltage dependent Ca^2+^ channels (VDCCs). Using Ca^2+^ imaging and electrophysiology, we searched for preNMDARs by uncaging NMDAR agonists either broadly throughout the whole field or locally at specific axonal locations. Releasing either NMDA or glutamate in the presence of NBQX using short laser pulses elicited current transients that were highly sensitive to the location of the spot and restricted to a small number of varicosities. The signal was abolished in the presence of high Mg^2+^ or by the addition of APV. Similar paradigms yielded restricted Ca^2+^ transients in interneurons loaded with a Ca^2+^ indicator. We found that the synaptic effects of NMDA were not inhibited by blocking VDCCs but were impaired in the presence of the ryanodine receptor antagonist dantrolene. Furthermore, in voltage clamped cells, bath applied NMDA triggers Ca^2+^ elevations and induces neurotransmitter release in the axonal compartment. Our results suggest the existence of preNMDARs in developing MLIs and propose their involvement in the NMDA-evoked increase in GABA release by triggering a Ca^2+^-induced Ca^2+^ release process mediated by presynaptic Ca^2+^ stores. Such a mechanism is likely to exert a crucial role in various forms of Ca^2+^-mediated synaptic plasticity.

## Introduction

In the central nervous system, postsynaptic NMDARs are often seen as canonical coincidence detectors in induction of synaptic plasticity. In addition, several lines of evidence indicate that NMDARs are present on presynaptic elements arguing for their possible involvement in presynaptic plasticity processes [1], [2]. Presynaptic NMDARs (preNMDARs) have been anatomically or functionally detected at both GABAergic [3]–[6] and glutamatergic [7], [8] termini (see [9] for review). Moreover, their implication in long-term plasticity has been suggested in various structures including the visual cortex [6], the neocortex [10], [11] and the cerebellum [8], [12], [13]. In the cerebellum, Lonchamp et al. [14] have shown that the preNMDARs expressed in parallel fibers (PFs) could mediate an increase in the frequency of mIPSCs recorded in Purkinje cells. On the other hand, several studies have suggested that MLIs express preNMDARs. Application of exogenous NMDA increases the frequency of miniature IPSCs (mIPSCs) [3], [12], [15] following an elevation of presynaptic Ca^2+^ [16]. This signal is thought to be provided by Ca^2+^ influx through preNMDARs rather than by activation of voltage-dependent Ca^2+^ channels (VDCCs) [4], [17], and to be amplified through a Ca^2+^-induced Ca^2+^ release (CICR) process [4]. Two main sources of glutamate have been proposed to activate preNMDARs on MLIs: spillover triggered by an intense stimulation of parallel fibers (PFs) [12], [18] or retrograde signaling from Purkinje cell dendrites [4], [19]. In cerebellar slices, the tonic activation of preNMDARs [3], [15] has been attributed to a spillover mechanism that is likely to be physiologically limited by glial glutamate transporters [15]. Moreover, glutamate spillover was proposed to contribute to long term potentiation of GABAergic synapses through preNMDAR stimulation [12] whereas retrograde activation of preNMDARs has been proposed to potentiate GABA release at the MLI-Purkinje cell synapse [4].

Even though activation of NMDARs clearly affects GABA release, it was recently argued that MLIs may not express axonal NMDARs [20], [21]. It was suggested instead that NMDARs are exclusively expressed in the somatodendritic domain, and that the depolarization induced by somatodendritic NMDARs is transmitted to the axon compartment due to the passive properties of the axon cable, leading to presynaptic VDCC activation [22]. A similar mechanism may apply to other neurons as well, so that the very existence of functional presynaptic NMDARs in brain neurons remains controversial [23].

In the present work we show that NMDA superfusion leads to axonal Ca^2+^ elevations under somatic voltage clamp and we map the NMDA sensitivity over the entire cell using wide field and local uncaging of NMDAR agonists. These methods reveal discrete spots of high sensitivity in the axon domain indicating that MLI could contain functional preNMDARs. Finally, we reexamine the pharmacological properties of NMDA-induced mIPSC enhancement, finding little sensitivity to VDCC blockers. Altogether the data provide a characterization of the effects of preNMDARs activation on cellular current and Ca^2+^ signaling.

## Results

### Activation of Axonal NMDARs Under Somatic Voltage Clamp Conditions

**Figure 1:**
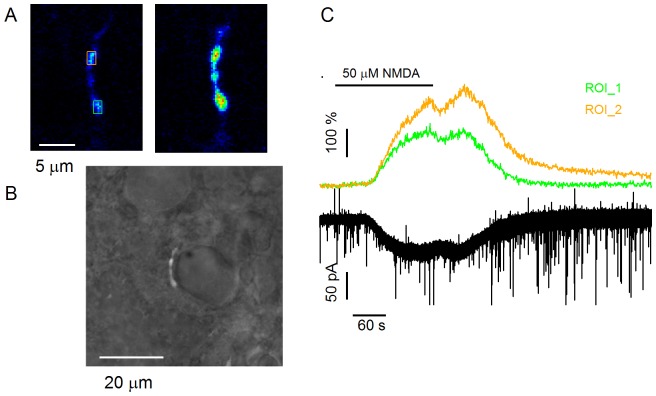
Presynaptic Ca^2+^ elevation in response to bath application of NMDA in a voltage clamped basket cell. The cell was voltage clamped at a holding potential of −60 mV in normal extracellular [Mg^2+^] (1 mM). It was perfused with the Ca^2+^ dye OGB-5N and part of its axon was imaged using 2-photon Ca^2+^ imaging. NMDA was added to the bath perfusion during the time indicated by bars (0.5 µM TTX throughout), while imaging a basket terminal belonging to the recorded cell. **A**: Fluorescence images from 2-photon laser scanning Ca^2+^ imaging at rest (left) and during the peak of the NMDA-induced current (right). **B**: Superposition of transmitted light and fluorescence images, showing that the fluorescent structure illustrated in **A** is a basket cell terminal in contact with a Purkinje cell soma. **C**: Time course of the NMDA-induced current and of the calcium-dependent fluorescent changes in 2 ROIs of the terminal as indicated by boxes in **A**, with matching colors in traces and in boxes.

The original suggestion that MLIs have functional preNMDARs has emerged from the observation that bath application of NMDA strongly increases the frequency of mIPSCs recorded in the two cell types that are innervated by MLIs i.e. Purkinje cells and other MLIs [3]. It was however immediately pointed out that, as an alternative or complementary interpretation to the direct activation of preNMDARs, axonal depolarization could result from activation of somatodendritic NMDAR following passive spread of somatic depolarization along the axon cable [3]. The latter component of the response would lead indirectly to GABA release, as the secondary depolarization of synaptic terminals would locally activate VDCCs producing an elevation of GABA release. Such a mechanism was recently described in the case of subthreshold somatic depolarisations [22], [24]. In fact, Christie and Jahr (2008, [20]) proposed that the NMDA-evoked depolarization exclusively arises from this indirect mechanism, and does not involve a direct action of NMDA on the axon. In this view, all NMDARs in MLIs would be somatodendritic. According to this hypothesis, somatic depolarization is an obligatory intermediate between activation of somatodendritic NMDARs and the subsequent increase in axonal Ca^2+^ concentration. If this is the case, then clamping the soma at a negative holding potential should prevent the effect entirely [20]. We therefore examined local axonal Ca^2+^ signalling following bath application of NMDA by performing 2-photon imaging of axon stretches in voltage clamped MLIs. In each experiment the identification of the recorded neurite as the axon was confirmed by analysis of action potential induced Ca^2+^ transients, which are markedly larger in the axon than in dendrites [25]. Following this identification, TTX (0.2 µM) and NMDA (50 µM) were sequentially added to the bath. In 7 MLIs, NMDA elicited inward currents with an average peak value of 26±8 pA under somatic voltage clamp (holding potential, −60 mV). Parallel to these currents, the Ca^2+^-dependent fluorescence in axonal varicosities increased in all experiments, with ΔF/F0 peak values ranging from 12 to 177% (63±10%, 19 varicosities from 7 cells). The fluorescence changes were transient, and returned to baseline occurring within 2 to 4 minutes after washing the agonist. The two largest Ca^2+^ transients in this series of experiments were obtained in basket cell terminals onto Purkinje cell somata. Fig. 1 illustrates one of these experiments, in which a basket cell was challenged with NMDA while imaging boutons on the Purkinje cell layer (Fig. 1A–C). The NMDA-induced current was accompanied by robust Ca^2+^ transients in the boutons directly in contact with the Purkinje cell soma. These data indicate that NMDA application is able to elicit large Ca^2+^ transients in basket cell terminals in spite of somatic voltage clamp.

In 5 additional experiments, NMDA applications were performed in the presence of 6 mM external Ca^2+^. The Ca^2+^-dependent fluorescence changes were larger with a peak ΔF/F0 amplitude of 340±89% (n = 5 cells). The effect had a gradual onset: even though a Ca^2+^ increase was observed as soon as NMDA was applied, the response typically grew over a time course of several minutes in the continuous presence of NMDA, and it declined rather slowly after washing out the NMDA, with a time for returning to baseline of 2 to 5 min after agonist removal. Such slow kinetics are consistent with a participation of intracellular Ca^2+^ stores in the NMDA-induced Ca^2+^ response.

In summary, bath application of NMDA elicits significant axonal responses under somatic voltage clamp, both with 2 mM and with 6 mM extracellular Ca^2+^. The axonal Ca^2+^ response cannot be attributed to voltage escape following activation of somato-dendritic NMDARs since the somatically recorded NMDA-induced currents in these experiments did not exceed 50 pA. Thus, somatic voltage clamp must have prevented depolarization spread from the dendrites to the axon. The axonal Ca^2+^ rise cannot be attributed to Ca^2+^ diffusion from the somatodendritic compartment since instead of the continuous concentration gradient expected from a diffusion process, a patchy response pattern was observed, with high response locations (which were often found in basket cell terminals) following much lower response locations along the axon course. Therefore these results, like those of similar experiments previously reported on stellate cells [15] are potentially difficult to reconcile with the indirect hypothesis.

### Wide Field Glutamate Photorelease

The preceding experiments leave the possibility open that NMDA would not bind directly to the MLI axon displaying the Ca^2+^ response, but to some other structure (not necessarily in the same cell), which would lead to the axonal response by unknown intermediary steps. Bath NMDA applications are not appropriate in this context since they lead to protracted activation of NMDARs with an imprecise timing. By contrast uncaging experiments provide a much more precise control of NMDAR activation timing. In the next series of experiments, we produced step increases in glutamate concentration by adding the photosensitive precursor of glutamate MNI-glutamate and by applying UV flashes to the entire preparation. Slices were incubated in a low Mg^2+^ (0.1 mM) HEPES-buffered saline supplemented with MNI-glutamate (final concentration, 0.9 mM) for at least 0.5 h before the beginning of the experiment. MLIs were perfused with Oregon Green BAPTA 1 (OGB-1) through the patch pipette and kept in the presence of NBQX (5 µM) to block AMPARs. Alexa 488 was added to resolve long axonal stretches. Ca^2+^ imaging was performed using a CCD camera (see methods).

**Figure 2:**
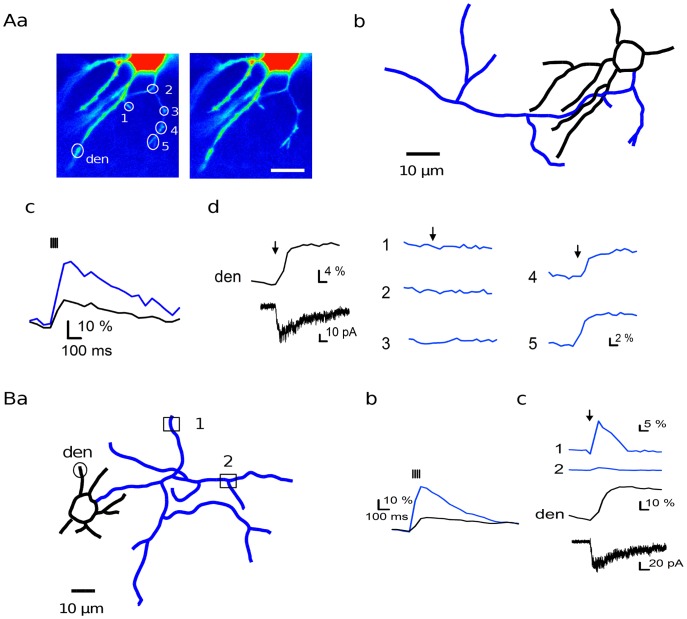
Wide field uncaging of MNI-glutamate reveals presynaptic NMDARs. **A**: MNI-glutamate was uncaged in a wide area of the recording chamber by a UV-flash through the microscope objective (see Methods) in 0.1 mM [Mg^2+^] and in the presence of both TTX (0.5 µM) and NBQX (5 µM). Fluorescence levels are shown before (**a, left**) and after (**a, right**) the flash. **b**: Reconstruction of the MLI (somatodendritic compartment in black and axon in blue). **c**: Ca^2+^ transients recorded before TTX application in response to 4 propagated action potentials in an axonal region (blue, region 5 in a) and in a dendritic region (black, region indicated «den» in a). **d**: MNI-glutamate uncaging in the presence of TTX and NBQX evoked a NMDAR-mediated current (left bottom trace) and Ca^2+^ transients in dendrites (left upper trace) as well as in axonal areas 4 and 5. **B**: Another experiment following the same experimental paradigm. **a**: Cell morphology. **b**: Responses to 4 propagated action potentials obtained before TTX application in axonal area 1 and in a dendritic location. **c**: Global uncaging of MNI-glutamate in TTX elicited Ca^2+^ transients both in the axonal and in the dendritic compartment (upper blue and black traces respectively), together with an inward current (bottom).

As depicted in Fig. 2Aa (left panel) the plane of focus typically contained several regions of interest that were selected according to their presumptive axonal or dendritic nature. A train of 4 propagated action potentials led to larger Ca^2+^ transients in the axonal compartment and therefore confirmed the identification of axon and dendrites (compare blue and black traces in Fig. 2Ac and Fig. 2Bb).

After application of TTX, a UV flash yielded somatic currents (36±4 pA, n = 23; Fig. 2Ad and Fig. 2Bc) accompanied by Ca^2+^ transients in dendrites (black traces in Fig. 2Ad and Fig. 2Bc; ΔF/F0∶33.7±5.0%, n = 11) as well as in certain regions of the axon (Fig. 2Ad, blue traces 4 and 5; Fig. 2Bc, blue trace 1; ΔF/F0∶11.2±1.7%, n = 13). Responsive axonal varicosities were found in 5 out of 6 cells. As discussed above, in the context of the experiments with bath application of NMDA, since the soma was under voltage clamp the only mechanism that could account for a participation of somatodendritic NMDARs to any axonal response would be Ca^2+^ diffusion along the axon. This can however be ruled out since responsive distal axonal spots follow irresponsive proximal spots as illustrated in Fig. 2Ad. In the case of the diffusion hypothesis, the Ca^2+^ concentration would be expected to gradually decrease from the soma. Importantly, axonal responses rose as fast as dendritic ones, with a delay shorter than the time resolution of imaging (50 ms). This argues against the possibility of slow intermediate steps intervening between NMDAR activation and axonal Ca^2+^ elevation.

### Local Glutamate Photorelease in the Axon

**Figure 3:**
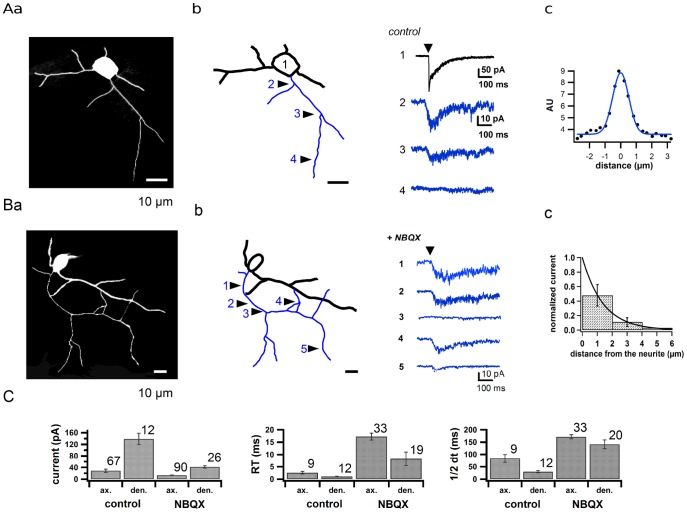
Activation of presynaptic ionotropic glutamate receptors using local glutamate uncaging. **A**: Responses obtained in TTX in the absence of NBQX. **a**: Fluorescence image of a MLI filled with 40 µM Alexa 488 via the patch pipette. **b**: Current responses to 0.3 ms-long laser ﬂashes that locally delivered glutamate from its photolabile precursor MNI-glutamate (1 mM) in the soma (1, black trace) and at various axonal locations (blue). **c**: The size of the actual uncaging spot was measured by drawing a line accross its image in fluorescence and fitting the projection of the spot on the line by a Gaussian curve (AU: fluorescence arbitrary units; full width at half maximum  = 1.38 µm). **B**: A similar experiment performed in the presence of NBQX (5 µM). **a**: Fluorescence image. **b**: Current responses to 1 ms-long laser pulses at various locations indicated by numbers on the cell reconstruction. **c**: Spatial resolution of axonal uncaging using MNI-glutamate in the presence of NBQX. To construct this curve the relative location of the laser and of the preparation was moved in the direction orthogonal to the neurite, in 4 separate experiments. The data were fit to an exponential decay with a space constant of 1.40 µm. **C**: Average amplitude (left), 20–80% risetime (middle) and half-decay time (right) of the currents evoked in dendrites (den) and axons (ax) by local uncaging of MNI-glutamate in TTX, in the absence (control) or in the presence (NBQX) of NBQX (5 µM). Error bars indicate ± sem; associated numbers indicate numbers of current traces contributing to the mean. All these experiments were performed in low extracellular [Mg^2+^] (0.1 mM).

To further characterize the local sensitivity of MLI axons to NMDA, we restricted photorelease of glutamate by using focused laser illumination [26]. In these experiments, like before, action potentials were blocked by TTX, and the local sensitivity of MLI neurites to glutamate was examined before and after blockage of AMPA receptors, using short and sharply focused laser pulses (405 nm; 0.1 to 1 ms). As shown in Fig. 3Ac, the uncaging spot had a half width of ∼1 µm. Releasing glutamate in the absence of AMPA receptor blocker elicited somatic current transients that differed markedly according to the location of the laser spot (Fig. 3A,B). Previous work suggest that AMPA receptors are present in both somatodendritic and axonal compartments of MLIs [27], [28], so that these experiments were expected to activate a mix of AMPA and NMDA receptors in both cases. When applied to the somato-dendritic compartment, laser pulses evoked robust current responses with rather high amplitude (139±19 pA, n = 12 locations out of 12 cells; Fig. 3A and C) and fast kinetics (20/80% rise time: 1.0±0.1 ms, n = 12; half decay time: 30±4 ms, n = 12; Fig. 3C). Axonal responses obtained under the same conditions displayed a smaller amplitude (28±6 pA, n = 67 varicosities out of 16 cells; Fig. 3A and C) and slower kinetics (20/80% rise time: 2.6±0.5 ms, n = 9 responses from 9 cells; half decay time: 84±16 ms, n = 9 responses from 9 cells; Fig. 3C). The somatodendritic responses were often biphasic and displayed a fast component with a time constant of decay smaller than 5 ms (Fig. 3Ab, black trace), presumably mediated by AMPARs. To isolate the contribution of NMDARs, the same experiments were performed in the presence of NBQX (5 µM). Somatodendritic responses were then found in every cell, however axonal responses were detected in only 12% of the cells (24 out of 202 cells). In responsive cells laser pulses applied along the axons gave rise to detectable currents in 28% of the locations. Uncaging of MNI-glutamate on dendritic spots gave rise to currents with an average amplitude of 42±4.2 pA (n = 26 locations out of 14 cells) with a 20/80% rise time of 8.3±2.8 ms (n = 19 out of 8 cells) and a half decay time of 141±18 ms (n = 20 out of 9 cells). The same experiments performed in the axon elicited currents displaying an average amplitude of 13±1.2 pA (n = 90 varicosities out of 24 cells), a 20/80% rise time of 17±1.4 ms (n = 33 responses out of 7 cells) and a half decay time of 172±8 ms (n = 33 responses out of 7 cells).

Axon identification is unambiguous in MLIs; however it could be envisaged that the responses that were attributed to axonal NMDARs were in fact elicited in dendritic structures that happened to be close enough to the targeted axon to be activated by glutamate diffusing away from the release spot. To address this possibility, we assessed the spatial resolution achieved by laser spot uncaging of MNI-glutamate with 1 ms pulse duration in the presence of NBQX (Fig. 3Bd). We found a very abrupt drop in sensitivity when moving the laser spot location away from the neurite, with a length constant of 1.4 µm. In view of these controls sensitive axonal spots that were located within 5 µm from any potential somatodendritic structure were rejected from the above analysis.

These experiments demonstrate that sizable current responses can be elicited from both axonal and dendritic locations following local and short activation of either AMPARs or NMDARs. The results reveal quantitative differences depending on neurite type and pharmacological conditions. Responses are larger and faster for AMPARs than for NMDARs, in accordance with the slower opening kinetics of NMDAR-associated channels than AMPAR-associated channels. Response onsets are also slower for axonal responses than for dendritic responses, as expected from the filtering effect of the axon cable. Nevertheless axonal responses delays are <10 ms suggesting that axonal responses implicate NMDARs that are locate in the axon and not in neighboring cell structures.

Reasons for the relative scarcity of NMDAR-mediated responses to local glutamate uncaging in axonal sites compared to the more reliable axonal responses found with wide field uncaging will be discussed below.

### Pharmacological Properties of Axonal NMDARs

While the above experiments strongly suggest the presence of functional NMDARs in the axon they could still be interpreted as reflecting the activation of non NMDA glutamate receptors following displacement of NBQX from the receptor binding site by released glutamate. Therefore it was important to investigate whether the presumptive axonal NMDAR-mediated responses displayed features expected from NMDARs. Three characteristic features of NMDA-gated channels were investigated: Mg^2+^ block, block by APV, activation by NMDA.

**Figure 4:**
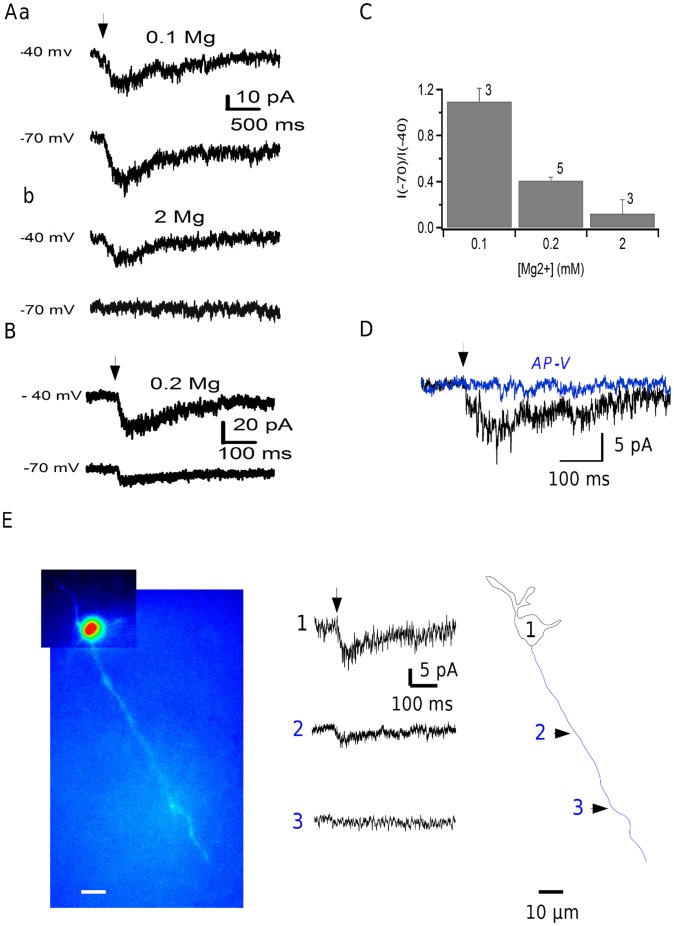
Features of NMDAR-mediated axonal responses using local uncaging. **A**: Responses to MNI-glutamate uncaging (arrow; AMPA receptors blocked with 5 µM NBQX; 0.5 µM TTX) at −40 and −70 mV are compared in the same axonal location in low external [Mg^2+^] (100 µM, upper traces) and in 2 mM Mg^2+^ (lower traces). **B**: Separate recording in 200 µM Mg^2+^, showing weakly voltage-dependent responses. **C**: Summary results. Numbers next to error bars (± sem) indicate the numbers of cells contributing to the means. **D**: Addition of AP-V (50 µM; blue) abolishes the uncaging response in the presence of NBQX (5 µM). **E**: Uncaging experiments using the novel cage MNI-NMDA. Left: Fluorescence view of a MLI filled with Alexa 488 through the patch pipette. Middle: Current traces obtained with focused 1 ms-long laser pulses delivered in the presence of MNI-NMDA (1 mM) at various locations indicated by numbers. Right: Reconstruction of the MLI with somatodendritic compartment in black and axon in blue.

Because of the well known blocking action of external Mg^2+^ ions on NMDA-sensitive channels [29] we performed experiments in a low external Mg^2+^ saline ([Mg^2+^] ∼0.1 mM). Under these conditions, the currents observed in response to axonal MNI-glutamate uncaging in the presence of NBQX were weakly dependent on the holding voltage (Fig. 4Aa,C). However, when Mg^2+^ was added to the bath, the currents became clearly voltage dependent, and started to display the characteristic outward rectification of NMDAR-coupled channels (Fig. 4Ab,C). Intermediate voltage dependence was observed for 200 µM Mg^2+^ in the bath (Fig. 4B,C).

Direct identification of the responses as arising from NMDARs activation was provided by the fact that when the specific NMDAR antagonist AP-V (50 µM) was added, the uncaging response was blocked (n = 3; Fig. 4D). Finally we investigated the sensitivity of the axonal channels to NMDA by using a novel NMDA cage instead of MNI-glutamate. Fig. 4E shows current traces obtained by local uncaging of MNI-NMDA (1 mM) in response to 1 ms laser pulses at various locations as indicated. Axonal current responses displaying an average amplitude of 11.5±3.5 pA were recorded in 6 cells out of a total of 29. Collectively, the results indicate that the axonal responses obtained by local MNI-glutamate uncaging in the presence of NBQX reflect the activation of NMDARs.

### Axonal Ca^2+^ Responses to Local NMDAR Activation

**Figure 5:**
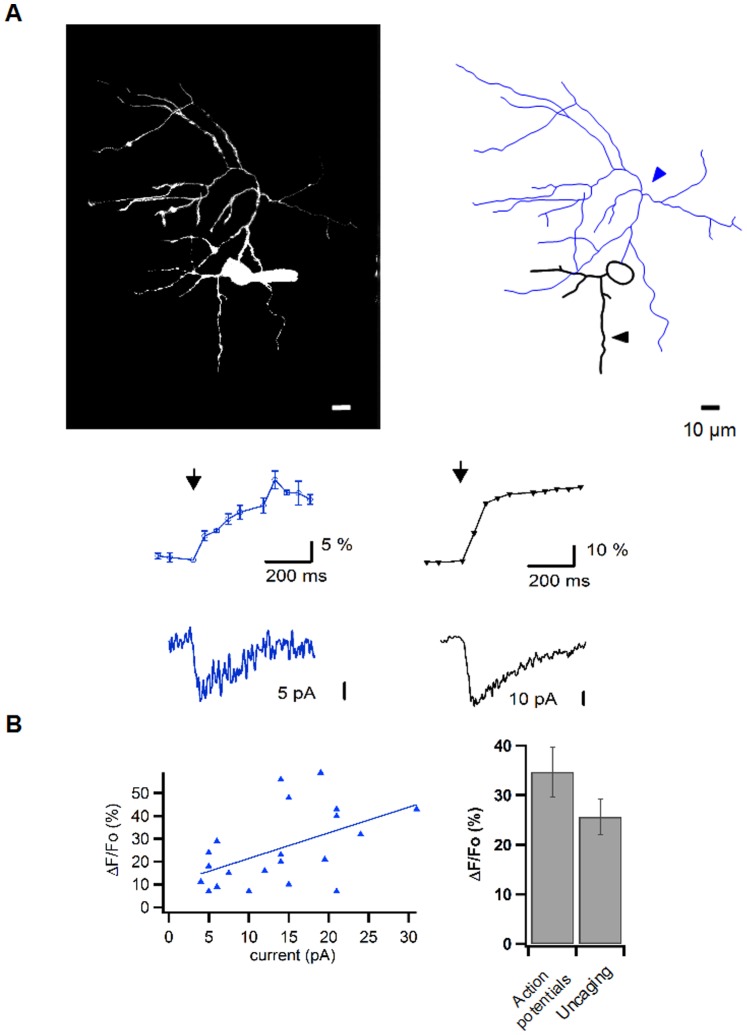
Axonal Ca^2+^ signals elicited by local activation of pre-NMDARs. **A**: Representative experiment. Top: Fluorescence image (left) and reconstruction (right) of a MLI filled with Alexa 488 (20 µM) and OGB-1 (50 µM). Bottom: Local Ca^2+^ transients and associated somatic currents obtained in response to axonal (left) and dendritic (right) glutamate release (1 ms laser pulses; 0.9 mM MNI-glutamate; 5 µM NBQX; 0.5 µM TTX). **B**: Summary data. Left: In responsive axonal spots, the amplitude of Ca^2+^ transients are correlated to the corresponding somatic current (correlation coefficient R = 0.74). Right: Ca^2+^ transients elicited in TTX by local glutamate uncaging (Uncaging) had peak amplitudes similar to those obtained in the same axonal spots before TTX application using 4 propagated action potentials (Action potentials). Experiments were carried out in normal extracellular [Mg^2+^] (1 mM).

Next we tested whether local axonal glutamate uncaging (Fig. 5A, blue arrowhead) could produce Ca^2+^ transients that would be associated with the current responses. Local MNI-glutamate uncaging in TTX gave rise to Ca^2+^ transients and to current responses both in the axonal (Fig. 5B, left) and in the dendritic domain (Fig. 5B, right). These transients rose with a time course of several hundreds of ms and they broadened during this time over a distance of ∼10 µm along the axon. Importantly, axonal spots that failed to deliver a current response also failed to exhibit a Ca^2+^ transient. In responsive spots, the amplitude of the axonal laser pulse-elicited Ca^2+^ transient (mean ΔF/F0, 25.6±0.8%, n = 21 out of 6 cells) was correlated to that of the current (mean, 13.8±0.4 pA, n = 21 out of 6 cells; Fig. 5C, left panel). As a rule, the Ca^2+^ transients elicited by uncaging had a similar amplitude as those obtained using 4 propagated action potentials before TTX application (respectively ΔF/F0 = 25.6±0.8%, n = 21 and ΔF/F0 = 34.7±1.3%, n = 15; Fig. 5C; right panel). Therefore the results indicate that a local and short activation of NMDARs in an axon spot is able to induce a large enough Ca^2+^ transient to increase transmitter release in the corresponding axonal area.

### Pharmacological Profile of NMDA Effect on Miniature Current Frequency

**Figure 6:**
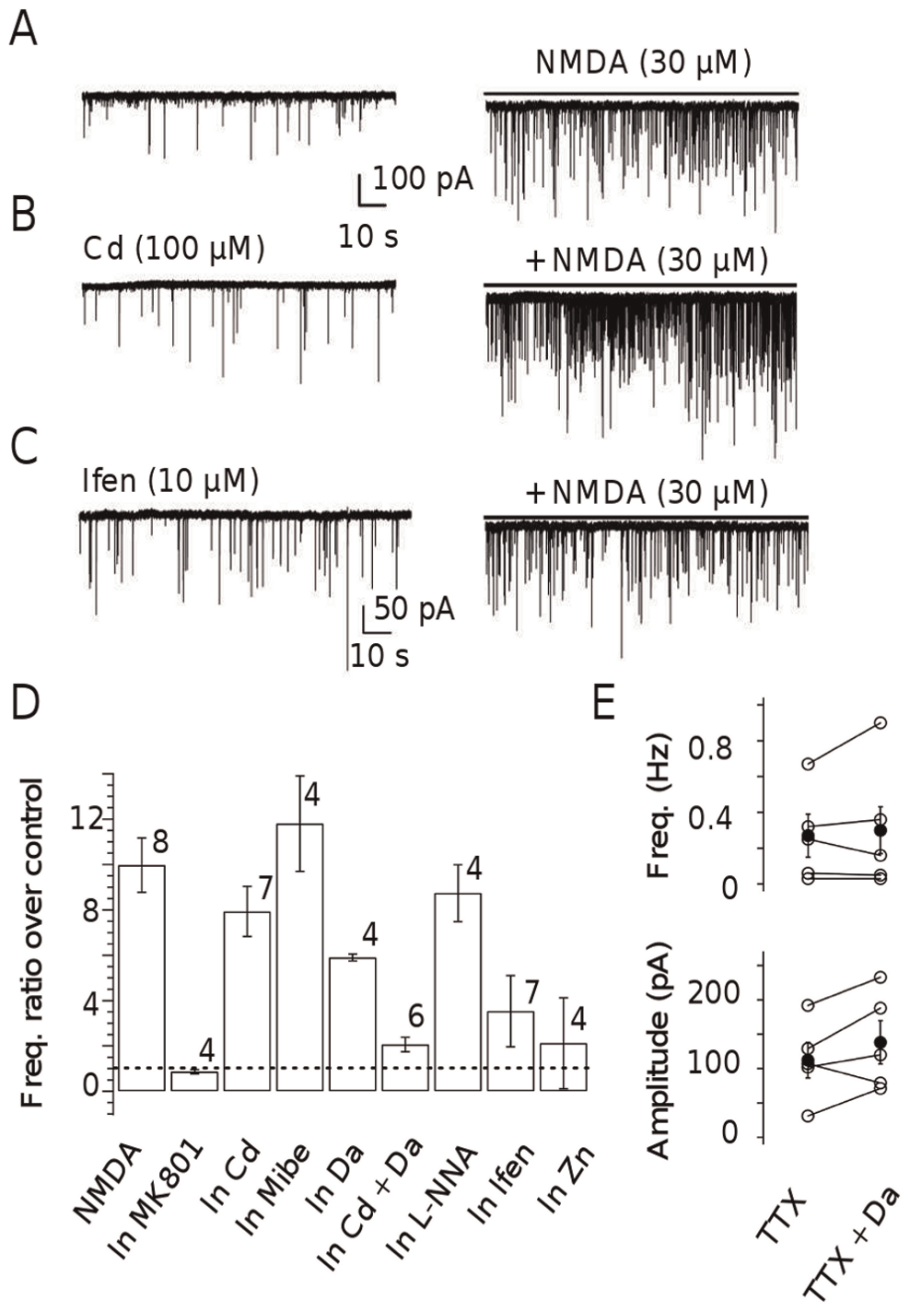
NMDAR activation increases the mIPSC frequency in MLIs. **A**: mIPSCs recorded from a representative MLI under control conditions (0.5 µM TTX, left) and in the presence of 30 µM NMDA (right). A modest inward current shift (control level indicated by continuous line on the right) represents activation of somatodendritic NMDARs. Note the marked mIPSC frequency increase. **B**, **C**: Similar experiments were performed in the continuous presence of Cd^2+^ (100 µM; **B**) or ifenprodil (10 µM, Ifen, **C**). **D**: Summary data. The percentage of increase of mIPSC frequency obtained with NMDA is plotted in various conditions: no drug (NMDA), MK801 (50 µM), Cd (Cd^2+^, 100 µM), Mibe (mibefradil, 10 µM), Da (dantrolene, 10 µM), Cd+Da (Cd^2+^ and dantrolene), L-NNA ((L)N-nitroarginine, 10 µM), Ifen (ifenprodil, 10 µM) and Zn (Zn^2+^, 300 nM). **E**: The presence of dantrolene (10 µM) does not significantly affect the frequency or the amplitude of mIPSCs. All experiments were carried out in normal extracellular [Mg^2+^] (1 mM).

The previous sections have examined the ability of glutamate to produce NMDAR-mediated current and Ca^2+^ responses in MLI axons. An alternative way to characterize the mechanism of action of NMDA is to examine the pharmacological profile of NMDA effect on miniature current frequency. In keeping with the pioneer study by Glitsch and Marty [3], we found that bath application of NMDA (30 µM) in the presence of TTX increased the frequency of mIPSCs with a frequency ratio over control of 9.97±1.19 (n = 8; Fig. 6 A and D). This increase was totally prevented by the presence of MK801 (50 µM) in the bath (ratio over control: 0.86±0.09; n = 4; Fig. 6D). Note that in MLIs, mEPSCs have a much lower frequency than mIPSCs, and that the two types of currents can be unambiguously distinguished on the basis of their decay kinetics [30]; therefore mEPSCs could easily be eliminated from the analysis and pharmacological block of AMPARs was not necessary in these experiments.

A simple approach that can be envisaged to distinguish between direct and indirect mechanisms of NMDA action is a pharmacological block of VDCCs. In the direct mechanism, NMDAR activation in the axon is likely to provide a Ca^2+^ rise even after total VDCC block, thus leading to mIPSC enhancement; in the indirect mechanism however, the response should be abolished by VDCC blockers. Therefore, we assessed the effects of NMDA on mIPSC frequency in the presence of 100 µM CdCl_2_ to block high threshold VDCCs. Under these conditions, the mIPSCs frequency ratio over control reached 7.93±1.10 (n = 7) indicating that high threshold VDCCs are not required for the response (Fig. 6 B and D). The increase in mIPSCs frequency was not significantly different in Cd^2+^ and in control (*P*>0.05; unpaired t-test) although Cd^2+^ ions have been reported to partially block NMDARs [31]. Low threshold VDCCs are not affected by Cd^2+^ ions and are expressed in MLIs [32]. The T-type channel blocker mibefradil (10 µM; [33]) did not alter the potentiating effect of NMDA on mIPSCs frequency (ratio over control : 11.8±2.1, n = 4; *P*>0.05; unpaired t-test; Fig. 6D). Taken together, these results concur with those previously obtained on Purkinje neurons [3], [17] to indicate that neither high threshold nor low threshold VDCCs are required for the response. They indicate that, contrary to the indirect depolarization theory, VDCC opening is not an obligatory step leading to the increase in GABA release during NMDA application.

Ryanodine-sensitive Ca^2+^ stores have been proposed to participate in the presynaptic Ca^2+^ elevation triggered by preNMDARs activation [4]. In line with this proposal, addition of dantrolene (10 µM), a muscle relaxing agent acting on ryanodine receptors, significantly reduced the potentiating effect of NMDA on mIPSC frequency (ratio over control : 5.9±0.15, n = 4; *P*<0.05; unpaired t-test; Fig. 6D). An even stronger effect was obtained by combining dantrolene and Cd^2+^ together (ratio over control : 2.1±0.32, n = 4; *P*<0.05; unpaired t-test; Fig. 6D). These results indicate that even though VDCCs are not required for the NMDA response, they contribute together with ryanodine-sensitive Ca^2+^ stores to enhance the response. Such an effect could reflect a functional coupling between VDCCs and the ryanodine receptor. Note that dantrolene alone neither affects the frequency (0.30±0.13 vs. 0.27±0.12 Hz, n = 5; *P*>0.05, paired t-test; Fig. 6E, upper panel) nor the amplitude of mIPSCs (138±31 vs. 112±26 Hz, n = 5; *P*>0.05, paired t-test; Fig. 6E, lower panel). In summary, intracellular Ca^2+^ stores do not participate in the regulation of the resting Ca^2+^ concentration but that they amplify the response to NMDA.

MLI axons contain neuronal NO synthase (nNOS; [34]), and MLI axon terminals have been suggested to engage an NMDAR-driven NO cascade during cerebellar LTD [16]. However NMDARs and nNOS have also been suggested to be located in PFs [8] so that local activation of NMDARs in PFs could lead to NO release and possibly to an indirect effect on MLI terminals that would be mediated by NO rather than by direct activation of NMDARs. In the presence of the nNOS antagonist L-NNA (10 µM). Under these conditions, NMDA still increased mIPSC frequency in a way that was not significantly different from that observed in control conditions (frequency ratio over control of 8.74±1.25, n = 4; *P*>0.05; unpaired t-test; Fig. 4D). These results indicate that the NMDA-induced enhancement of GABA release in MLIs is due to the activation of NMDARs located in MLI axons rather than to an indirect mechanism involving NO release from neighboring structures.

The effects of NMDA on synaptic activity were readily observed in normal TTX- containing BBS. This indicates that the preNMDARs activation occurs even in the presence of the normal Mg^2+^ concentration (1 mM) without any pre-depolarization to relieve the Mg^2+^ block. To explain this result, it has been postulated that preNMDARs could incorporate NR2C and D subunits in their structure which would render them less sensitive to Mg^2+^ inhibition than their NR1 and NR2A/B counterparts [3], [4]. Alternatively, axonal depolarization could amplify preNMDAR activation. Zn^2+^ ions are widely used to distinguish NR2A subunits as they appear to be far more selective for NR1/NR2A receptors than for any other NR2 subunit-containing receptors [35]. In the presence of 300 nM Zn^2+^, NMDA (30 µM) did not significantly increase the frequency of mIPSC (frequency ratio over control of 2.12±2.00, n = 4; Fig. 6D). Additionnally ifenprodil, an NR2B selective antagonist, was used to examine the possible involvement of this subunit in the effect of NMDA application. When slices were treated with 10 µM ifenprodil, application of NMDA (30 µM) still increased the frequency of mIPSCs (frequency ratio over control of 3.53±1.57; Fig. 6 C and D), albeit to a smaller extent than in control conditions. These results indicate that both NR2A and NR2B participate in the effects elicited by NMDA on mIPSCs. Because of the high input resistance of MLIs and the rather small volume of their presynaptic terminals, even a small glutamate-evoked current might be sufficient to alleviate Mg^2+^ block. It is important to mention that Zn^2+^, ifenprodil, mibefradil, L-NNA do not alter mIPSC frequency by themselves.

### NMDA Application Increases the Frequency of Preminis

The results of Fig. 1 show that under somatic voltage clamp conditions, where the indirect mechanism of NMDA-induced GABA release is blocked, activation of axonal NMDARs leads to a large elevation of the cytosolic Ca^2+^ concentration. To test whether this effect was able to increase neurotransmitter release, we examined a new class of miniature currents referred to as « preminis » [36]. Preminis reflect the activation of presynaptic GABA_A_Rs through individual vesicular release events and therefore require neurotransmitter release from the recorded cell. They exhibit specific amplitude and kinetics that set them apart from conventional miniature events.

**Figure 7:**
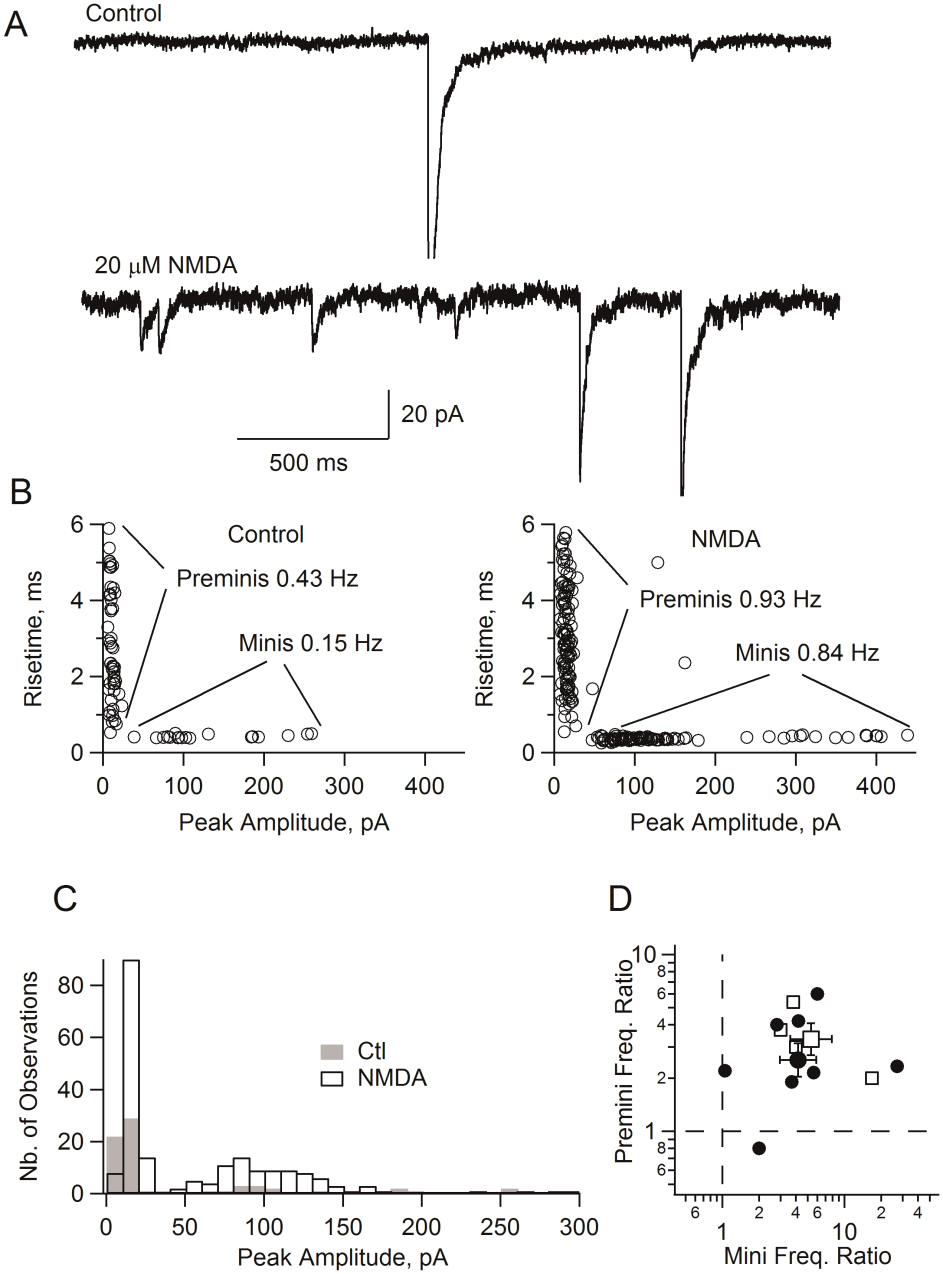
NMDA application increases premini frequency in voltage-clamped MLIs. **A**: Spontaneous synaptic currents in the presence of TTX (preminis are identified as events having a peak amplitude of <30 pA; there is 1 such event in the upper trace, plus one somatodendritic miniature) and after further bath application of 20 µM NMDA (lower trace: 5 preminis plus 2 minis). The soma is voltage clamped at −60 mV. 2 of the miniature events, one in control and one in NMDA, have their peaks clipped off. **B**: Plots of 20–80% risetime as a function of peak amplitude in control (2 min duration) and in NMDA (also 2 min), from the same experiment as in **A**. Conventional miniature currents (minis) appear as a cluster of events with peak amplitudes >30 pA and risetimes <0.7 ms, and presynaptic miniature currents (preminis) appear as a non overlapping cluster of events with peak amplitudes <30 pA and risetimes >0.5 ms. Note that the frequencies of both preminis and minis increase in response to NMDA application. **C**: Peak amplitude histograms from the data in **B** (gray: control; open bars: NMDA). **D**: *Dots*: summary of 8 experiments (5 with 20 µM NMDA, and 3 with 50 µM NMDA), showing ratios of both premini and mini frequencies in NMDA over control periods. Only 1/8 experiment fails to show an increase in the premini frequency in response to NMDA. *Open squares*: summary of 4 experiments performed in the presence of NBQX (20 µM), Cd^2+^ (100 µM) and NMDA (50 µM). Experiments have been carried out in normal extracellular [Mg^2+^] (1 mM).

A representative experiment is illustrated in Fig. 7. Under control conditions (TTX, 0.5 µM) preminis had a peak amplitude smaller than 30 pA and risetime values ranging from 0.5 to 6 ms (Fig. 7A, B). By contrast conventional miniature events had amplitudes of 30–300 pA and homogeneous risetime values of about 0.4 ms (Fig. 7A, B). In the presence of NMDA, the recording noise increased, but it was still possible to identify the two separate classes of events, and the frequencies of both preminis and conventional miniatures increased (respectively from 0.43 to 0.93 Hz, and from 0.15 to 0.84 Hz; Fig. 7 A–C). Summary results from 7 cells gave a mean increase of 2.5-fold for preminis, and of 4.1-fold for conventional miniature currents (Fig. 7D).

Like the previous results of Fig. 6, the premini frequency increase must be due to activation of preNMDARs because the soma was under voltage clamp. The results of Fig. 7 indicate that the elevation of the axonal Ca^2+^ concentration elicited by preNMDAR activation is able to induce transmitter release in spite of the somatic voltage clamp. To confirm that premini activation was not due to an indirect mechanism involving neighboring MLIs, mIPSCs and preminis were recorded in the presence of NBQX (20 µM) and Cd^2+^ (100 µM) to block the indirect pathway. In the presence of NMDA, the frequency of preminis increased from 0.12 to 0.46 Hz. Averaged results from 4 cells gave a mean increase of 3.3-fold for preminis and 5.2 for minis (Fig. 7D; open square symbols). These values are not significantly different from these obtained without blockers, thus confirming that the increase in premini frequency is due to activation of axonal NMDARs in the recorded cell.

It is worth noting that the augmentation of premini frequency is of the same order of magnitude as that of conventional miniatures (which originate from unclamped MLIs). The increase measured for preminis is actually smaller than that of conventional miniatures. However, the first value is an underestimate because NMDA decreases the length constant of the axon cable by increasing the membrane permeability of this cable, thus decreasing the effective surface area from which preminis are collected. Since this effect could easily explain the difference between the 2.5-fold increase in premini rate and the 4.1-fold increase in mini frequency, the results suggest that the entire scope of the NMDA-induced frequency increase can be accounted for by the direct activation of axonal NMDARs.

## Discussion

Altogether our results fail to substantiate an indirect mechanism for the responsiveness of MLI axons to NMDA, and suggest instead a direct mechanism reflecting the activation of NMDARs in the axonal domain of these cells. Here we briefly review the key new findings that favor the direct mechanism, and we proceed with a discussion of the specific features of axonal NMDAR-mediated signaling that emerge from the present work.

### NMDA-induced Axonal Ca^2+^ Rise and Subsequent GABA Release under Somatic Voltage Clamp

Clamping the somatic potential at a negative value did not prevent NMDA-induced axonal responses. Large axonal Ca^2+^ elevations were still elicited by NMDA application and furthermore, these Ca^2+^ elevations were able to elicit GABA release as demonstrated by the fact that the rate of preminis was significantly enhanced. Thus, contrary to the predictions of the indirect mechanism hypothesis, preventing somatodendritic NMDARs from depolarizing the axon compartment does not abolish the NMDA response.

### Uncaging Experiments

The results obtained with wide field uncaging confirm that most axons are responsive to NMDA, but they also indicate that this sensitivity is restricted to a small number of varicosities. In addition, they rule out Ca^2+^ diffusion from the somatodendritic compartment as an explanation for NMDA-induced axonal Ca^2+^ elevations under voltage clamp because no Ca^2+^ gradient from soma/dendrite to axon terminals was observed.

Local uncaging either of NMDA or of glutamate in conditions where both AMPA-selective and kainate-selective glutamate receptors are blocked, leads to the generation of an axonal current that can be collected in whole-cell recording. These experiments allow for a much more precise and reproducible agonist application than the previous approach based on iontophoresis [20], [21], but they nevertheless suffered from some technical limitations. First, axonal structures were not always available, since the axon was sometimes located under the recording pipette. In other cases the axon was visible but plundged into the slice tissue. Because the loss of 405 nm laser intensity due to scattering is approximately two-fold per 18 µm [26], the uncaging efficiency of local laser stimulation declines quickly with depth. This explains why responsive axonal stretches were immediately near the slice surface. Second, experience proved that only a limited number of laser flashes could be applied before photodamage was evident, so that a relatively small number of locations (typically not more than 5) were tested in a given experiment. These limitations explain that only a minority of uncaging experiments revealed any axonal sensitivity to NMDAR, without implying the existence of non responsive axons.

In responding axons, only 28% of the probed locations gave rise to a detectable response. This result is in keeping with the fact that in cultured MLIs, 39% of the patches excised from axon terminals did not display functional NMDARs [5] indicating that a significant proportion of varicosities does not contain NMDARs. The sparse nature of axonal NMDA signaling may account in part for the previous failure to detect axonal NMDARs using iontophoretic NMDA applications [20], [21], [37] as well a two-photon uncaging of MNI-glutamate [21]. The exact nature of the responsive spots remains to be determined but they presumably include basket terminals onto Purkinje cells since these structures display presynaptic NMDAR immunostaining [38] and since they appeared especially responsive to NMDA in the present work.

### PreNMDAR Activation Leads to Ca^2+^ Transients

In the presence of OGB-1 in the intracellular recording solution, we were able to simultaneously record Ca^2+^ transients and NMDA-evoked currents. The amplitudes of the transients obtained with 1 ms laser pulses were comparable to those of summed transients evoked by four action potentials in close succession. It seems therefore plausible that a short-lived activation of preNMDARs leads to a robust release of GABA. These transients were found to spread along the axon over a distance of ∼10 µm, possibly reflecting diffusion as well as the recruitment of ryanodine-sensitive intracellular Ca^2+^ stores [4]. Local amplification by intracellular Ca^2+^ stores may also account for the negative results obtained with aspartate iontophoresis by [20], since the constant use of the SERCA pump inhibitor cyclopiazonic acid in these experiments may have emptied Ca^2+^ stores. Taken together, our data indicate that this axonal sensitivity to NMDA is strong enough to fully account for the effects observed on miniature currents.

### Pharmacological Profile of the mIPSC Response to NMDA

According to the indirect mechanism the mIPSC frequency increase elicited by NMDA should be highly sensitive to VDCC blockers. However we found that neither the general VDCC blocker Cd^2+^, or the T-type VDCC blocker mibefradil, were able to inhibit this effect significantly. These results together with those of a previous study [17], demonstrate that activation of VDCC is dispensable for the mIPSC response. The strength of the inhibitions observed with NR2 subunit antagonists (i.e. Zn^2+^ and ifenprodil) indicate that the NR3A subunit is not present presynaptically unlike in the neocortex where NR3A-containing preNMDAR promote neurotransmitter release and spike timing-dependent plasticity [39]. Indeed, the expression of NR3A in cerebellum has been shown to be barely detectable after 10 days PN [40]. The half-decay time of preNMDARs current is consistent with mixed involvement of NR2A and -B subunits since it matches the deactivation kinetics of the currents recorded in HEK cells expressing recombinant NMDARs containing either NR2A or NR2B subunit [13].

### Functional Role of preNMDARs

In MLIs, activation of preNMDARs i) increases spontaneous and miniature IPSC frequency in Purkinje cells and MLIs; ii) triggers the onset of depolarization-induced potentiation of inhibition (DPI) in immature Purkinje cells [4] and iii) leads to I-LTP in MLIs [12]. Application of D-APV has been shown to slightly decrease the frequency of mIPSCs recorded in Purkinje cells [3], [15] suggesting a tonic activation of NMDARs. These results suggest that the regulation of IPSC frequency occurs as soon as the ambient glutamate concentration exceeds normal levels, whether as a result of some local unbalance or because of strong or sustained parallel fibre activity; because of the NMDAR-mediated increase in spontaneous inhibition on postsynaptic MLIs and Purkinje cells, the effects of ambient glutamate on the mean level of cell firing in the molecular layer are then kept within acceptable limits. On a somewhat longer time scale (minutes) the onset of DPI triggers a regulatory loop by which increased MLI inhibition corrects excessive firing of Purkinje cells. Finally I-LTP fulfills a similar role on a still longer time scale (hours). Therefore all three effects may be seen as contributing to reestablishing near-normal conditions in case of excessive neuronal activity.

The strong inhibition of miniature current frequency observed with a combination of VDCC and ryanodine receptor antagonists suggest that the activation of preNMDARs triggers a signaling module involving both VDCCs and Ca^2+^ stores in the amplification of the initial rise in presynaptic Ca^2+^. Moreover, the dectection of nNOS [34] and PSD-95 [41] immunoreactivity in MLI terminals led to propose the existence of NMDAR-containing signaling complexes [16]. Such units are likely to contribute to the NO-dependent form of LTP that is produced at the PF-stellate cell synapse by pairing low frequency stimulation of PFs with postsynaptic depolarization [42]. An NMDA/nNOS unit in MLI was also suggested to support the induction of LTD at the PF-PC synapse [16] although a similar cascade has been shown to directly take place in PFs [13]. The recruitment of the NMDAR-NOS Ca^2+^ signaling pathway in MLIs could simultaneous trigger LTD of PF-Purkinje cell synapses and LTP of PF-MLI synapses, a coincidence that may result in a strong attenuation of Purkinje cell activity.

## Materials and Methods

### Ethics Statement

Sprague–Dawley rats were purchased at Elevage Janvier (Saint Berthevin, France). They were housed at the animal house of Centre Universitaire des Saints Pères, which is approved by the ‘Préfecture de Police’ following inspection by Veterinary Services of the city of Paris, and representatives of the French Ministry of Research and the Ministry for Health, in agreement with the European Directive 86/609/EEC regarding the protection of animals used for experimental purposes (approval number A-750607).

### Slice Preparation

Postnatal day 12–16 rats were killed by decapitation under general anaesthesia following inhalation of the volatile anaesthetic isoﬂurane. Parasagittal (200 µm) cerebellar slices were cut with a vibroslicer (Leica VT1200S, Germany) in ice-cold bicarbonate buffered saline (BBS) solution containing the following (in mM): 125 NaCl, 2.5 KCl, 2 CaCl_2_, 1 MgCl_2_, 1.25 NaH_2_PO_4_, 26 NaHCO_3_, and 10 glucose (saturated with 95% O_2_ - 5% CO_2_), pH 7.3. The slices were incubated at ∼34°C for 30 min and were then stored at room temperature. During recordings, the slices were superfused with the above solution (1–1.5 ml/min) at room temperature (20–23°C) except for uncaging experiments.

### Stellate and Basket Cells

MLIs were identified using an upright microscope (Axioskop, Zeiss, Germany) with differential interference contrast (DIC) optics, a 60X Olympus objective, 0.90 NA water immersion objective, and a 0.63 NA condenser. In the present report, the same results were found in all interneurons tested irrespective of their location in the molecular layer, we therefore refer collectively to stellate and basket cells as MLIs.

### Recording and Analysis of IPSCs

MLIs were maintained under voltage clamp in the whole-cell recording (WCR) configuration at a holding potential of −70 mV. The intracellular solution contained (in mM) 150 KCl, 4.6 MgCl_2_, 0.1 CaCl_2_, 10 HEPES-K, 1 mM EGTA-K, 0.4 Na-GTP, and 4 Na-ATP, pH 7.3. Tight-seal WCRs were obtained with borosilicate pipettes (4–6 MΩ) from superficial somata using an EPC-9 amplifier (HEKA Electronik, Darmstadt, Germany). Series resistance values ranged from 15 to 25 MΩ and were compensated for by 60%. Currents were filtered at 1.3 kHz and sampled at a rate of 250 µsec/point. N-methyl D-aspartate (NMDA) was added to the bath at 30–50 µM. mIPSCs and “preminis” were recorded in the presence of 0.5 µM TTX. For pharmacological studies, synaptic current parameters were compared during a control period preceding NMDA application and a test period lasting from 2 to 3 min after onset of the application. Detection and analysis of IPSCs were performed off-line with routines written in the IGOR Pro programming environment (WaveMetrics). Note that in MLIs, most spontaneous synaptic currents were blocked by bicuculline (20 to 50 µM) and were therefore mediated by activation of GABA_A_ receptors. IPSCs and EPSCs could be distinguished based on the decay kinetics of the two currents which differ by an order of magnitude as reported [30]. For specific experiments, Zn^2+^ was buffered with tricine [N-tris(hydroxymethyl)methylglycine], as described [43].

### Wide Field Uncaging of MNI-glutamate

Whole field photolysis of MNI-glutamate was achieved using a pulsed xenon arc lamp (Till Photonics, Germany) as described [44]. Briefly, a high intensity (0.5 ms duration; 80 J) discharge of UV light (360±7.5 nm) was reflected onto the plane of focus using a dichroic mirror and the 60X water immersion objective. For uncaging experiments, the perfusion was turned off to minimize consumption of the cages. A HEPES-buffered solution supplemented with NaHCO_3_ to control internal pH was used (composition in mM: 135 NaCl, 4 KCl, 2 NaHCO_3_, 25 glucose, 2 CaCl_2_, 0.1 MgCl_2_ and 10 HEPES, pH 7.4 with NaOH).

### Local Uncaging of MNI-glutamate and MNI-NMDA

Laser photolysis was carried out by implementing a 405 nm diode laser (Point Source Iflex 2000) coupled with a single mode optical fibre into the epifluorescence condenser of a commercial microscope (Zeiss Axioskop FS1) as described previously [26]. In this implementation the fibre output of the laser was expanded with a 40 mm focal length positive lens in a dual LED lamphouse (OptoLED, Cairn Research, UK) and reflected with a 45° dichroic mirror (425 DCXR, Chroma) into the epifluorescence condenser of the microscope. The epifluorescence condenser was previously adapted to accommodate output of a TILL monochromator (Polychrome 2, TILL Photonics, Germany) and the final dichroic reflector changed to an extended UV type (T490LP, Chroma). After initial alignment, the laser fibre and first lens were adjusted on three axes to position and focus the uncaging spot in the microscope focus. A spot of approximately 1 µm diameter was formed at the focal plane in a 100 µM pyranine solution using a CCD camera (TILL Photonics, Germany). The straight-through path of the LED lamphouse received the fibre output of the monochromator for epifluorescence excitation at 488 nm (slit with 10 nm).

### Calcium Imaging

Two different setups were used for Ca^2+^ imaging experiments, either a custom two-photon laser-scanning microscopy (2PLSM) system ([45] and references therein) or a single-photon system. The 2PLSM setup was used to assess the axonal Ca^2+^ rise induced by bath application of NMDA. Other experiments were performed on a single photon setup from TILL Photonics (Germany) based on a Polychrome 2 monochromator and a Peltier-cooled CCD camera (IMAGO QE; 1376×1040 pixels; pixel size: 244 nm after 53X magnification and 2×2 binning). Excitation was at 480 nm and images were acquired with emission at 510–550 nm. To induce axonal [Ca^2+^]i rises, trains of 4 action potentials (20 ms intervals) were produced by depolarizing the cell for 3 ms to 0 mV from a holding value of −70 mV thus inducing a propagated action potential [46].

Whole-cell recording pipettes were filled with (in mM) 140 K-gluconate, 5.4 KCl, 4.1 MgCl_2_, 9.9 HEPES-K, 0.36 Na-GTP, 4 Na-ATP, pH was adjusted to 7.3 with KOH. For 2-photon laser scanning imaging experiments, this solution was supplemented with 400 µM Fluo 4 (Invitrogen). For single photon excitation, the solution contained 50 µM Oregon Green 488 BAPTA-1 (OGB-1; Invitrogen). and Alexa 488 fluor (20 µM; Invitrogen) was added for local uncaging to facilitates axon identification early in the experiments. However, ΔF/F0 values reported here for trains of action potentials (ΔF/F0 = 34.7±1.3%; n = 15; Fig. 5C) are lower than those previously reported for the axons using OGB-1 alone.

For all Ca^2+^ imaging experiments, neurites identification was confirmed by analysis of action potential induced Ca^2+^ transients, which are markedly larger in the axon than in dendrites as reported [25].

Analysis was performed by calculating the average fluorescence in regions of interest (ROIs) as a function of time as detailed before [45].

The following drugs applied in the bath were used when appropriate: N-methy-D-aspartate (NMDA), tetrodotoxin (TTX), Mibefradil, cadmium chloride (CdCl_2_), zinc chloride (ZnCl_2_), Ifenprodil, 2,3-dihydroxy-6-nitro-7-sulfamoyl-benzo[f]quinoxaline-2,3-dione (NBQX), DL-2-Amino-5-phosphonopentanoic acid (AP-V), Nω-nitro-L-arginine (L-NNA), dantrolene. All drugs were purchased at Ascent. Other chemicals were purchased at Sigma. 4-Methoxy-7-nitroindolinyl-caged-L-glutamate (MNI-Glutamate) and 4-Methoxy-7-nitroindolinyl-caged-NMDA (MNI-NMDA) were kindly provided by Tocris Bioscience.
